# The broad phenotypic spectrum of 17α-hydroxylase/17,20-lyase (CYP17A1) deficiency: a case series

**DOI:** 10.1530/EJE-21-0152

**Published:** 2021-09-15

**Authors:** Min Sun, Jonathan W Mueller, Lorna C Gilligan, Angela E Taylor, Fozia Shaheen, Anna Noczyńska, Guy T’Sjoen, Louise Denvir, Savitha Shenoy, Piers Fulton, Timothy D Cheetham, Helena Gleeson, Mushtaqur Rahman, Nils P Krone, Norman F Taylor, Cedric H L Shackleton, Wiebke Arlt, Jan Idkowiak

**Affiliations:** 1 Institute of Metabolism and Systems Research, College of Medical and Dental Sciences, University of Birmingham, Birmingham, UK; 2 Centre for Endocrinology, Diabetes and Metabolism, Birmingham Health Partners, University of Birmingham and University Hospitals Birmingham NHS Foundation Trust, Birmingham, UK; 3 Department of Endocrinology and Diabetology for Children and Adolescents, Wroclaw Medical University, Wroclaw, Poland; 4 Department of Endocrinology, Ghent University Hospital, Ghent, Belgium; 5 Department of Paediatric Endocrinology and Diabetes, Queen’s Medical Centre, Nottingham, UK; 6 Children’s and Adolescent Services, University Hospitals of Leicester NHS Trust, Leicester, UK; 7 West Midlands Regional Genetics Service, Birmingham Women’s and Children’s NHS Foundation Trust, Birmingham, UK; 8 Newcastle University c/o Department of Paediatric Endocrinology, Royal Victoria Infirmary, Newcastle Upon Tyne, UK; 9 Department of Endocrinology, University Hospitals Birmingham NHS Foundation Trust, Birmingham, UK; 10 Department of Endocrinology, Northwick Park Hospital, London Northwest University Healthcare NHS Trust, London, UK; 11 Academic Unit of Child Health, Department of Oncology & Metabolism, University of Sheffield, Sheffield, UK; 12 Department of Clinical Biochemistry, King’s College Hospital, London, UK; 13 Benioff Children’s Hospital, University of California San Francisco, Oakland, California, USA; 14 Department of Endocrinology and Diabetes, Birmingham Children’s Hospital, Birmingham Women’s and Children’s NHS Foundation Trust, Birmingham, UK

## Abstract

**Context:**

17α-Hydroxylase/17,20-lyase deficiency (17OHD) caused by mutations in the *CYP17A1* gene is a rare form of congenital adrenal hyperplasia typically characterised by cortisol deficiency, mineralocorticoid excess and sex steroid deficiency.

**Objective:**

To examine the phenotypic spectrum of 17OHD by clinical and biochemical assessment and corresponding *in silico* and *in vitro* functional analysis.

**Design:**

Case series.

**Patients and results:**

We assessed eight patients with 17OHD, including four with extreme 17OHD phenotypes: two siblings presented with failure to thrive in early infancy and two with isolated sex steroid deficiency and normal cortisol reserve. Diagnosis was established by mass spectrometry-based urinary steroid profiling and confirmed by genetic *CYP17A1* analysis, revealing homozygous and compound heterozygous sequence variants. We found novel (p.Gly111Val, p.Ala398Glu, p.Ile371Thr) and previously described sequence variants (p.Pro409Leu, p.Arg347His, p.Gly436Arg, p.Phe53/54del, p.Tyr60Ile*fs*Lys88X). *In vitro* functional studies employing an overexpression system in HEK293 cells showed that 17,20-lyase activity was invariably decreased while mutant 17α-hydroxylase activity retained up to 14% of WT activity in the two patients with intact cortisol reserve. A ratio of urinary corticosterone over cortisol metabolites reflective of 17α-hydroxylase activity correlated well with clinical phenotype severity.

**Conclusion:**

Our findings illustrate the broad phenotypic spectrum of 17OHD. Isolated sex steroid deficiency with normal stimulated cortisol has not been reported before. Attenuation of 17α-hydroxylase activity is readily detected by urinary steroid profiling and predicts phenotype severity.

**Significance statement:**

Here we report, supported by careful phenotyping, genotyping and functional analysis, a prismatic case series of patients with congenital adrenal hyperplasia due to 17α-hydroxylase (CYP17A1) deficiency (17OHD). These range in severity from the abolition of function, presenting in early infancy, and unusually mild with isolated sex steroid deficiency but normal ACTH-stimulated cortisol in adult patients. These findings will guide improved diagnostic detection of CYP17A1 deficiency.

## Introduction

The classic phenotypic presentation of 17α-hydroxylase deficiency (17OHD) comprises cortisol and sex steroid deficiency combined with mineralocorticoid excess (1). Affected genetic males (46,XY) commonly present with female external genitalia at birth, are usually raised as girls and are often not diagnosed until they reach adolescent age when they present with pubertal delay and hypertension, similar to 46,XX females. Patients typically escape the adrenal crisis, despite being profoundly cortisol deficient, due to the affinity of accumulating corticosterone for the glucocorticoid receptor (1, 2).

The molecular bases of 17OHD are inactivating mutations in the *CYP17A1* gene, coding for an enzyme that is located at a major branch point in human steroidogenesis. CYP17A1 (also P450c17) exhibits two distinct catalytic activities. CYP17A1 17α-hydroxylase activity is crucial for cortisol biosynthesis in the adrenal by converting progesterone and pregnenolone to 17-hydroxyprogesterone (17OHP) and 17-hydroxypregnenolone (17Preg), respectively, the 21-carbon precursors of cortisol. CYP17A1 17,20-lyase activity generates sex steroids in adrenal and gonads by converting 17OHP to androstenedione and, with about 50-fold higher efficiency (3), 17Preg to DHEA, the principal androgen precursor in humans. CYP17A1 requires electron transfer from NAD phosphate (NADPH). For sufficient reaction efficiency, the flavoprotein P450 oxidoreductase (POR) is crucial for both catalytic steps (4, 5) and the small haemoprotein cytochrome b5 (CYB5A) for efficient 17,20-lyase activity (6).

To date, more than 100 mutations in the *CYP17A1* gene have been described (www.hgmd.cf.ac.uk), and the majority are associated with a classic phenotype of combined 17α-hydroxylase/17,20-lyase deficiency. A smaller number of *CYP17A1* missense variants are reported to exhibit partial impairment of 17α-hydroxylase/17,20-lyase activity, where hypertension is mild or absent and external genitalia appear ambiguous in 46,XY individuals (7, 8, 9, 10, 11). A small number of individuals affected by 17OHD present with apparently isolated 17,20-lyase deficiency, with abolished or severely reduced 17,20-lyase activity but only mild to moderate impairment of 17α-hydroxylase activity (12, 13, 14). These patients had normal blood pressure and random cortisol levels but reduced sex steroid production. This suggests that variations in CYP17A1 17α-hydroxylase activity determine the width of the phenotypic spectrum of 17OHD.

We have analysed a series of eight patients with 17OHD including four patients with novel phenotypes at opposite ends of the phenotypic spectrum: two young siblings with indications of glucocorticoid deficiency at one end and two patients with isolated sex steroid deficiency and normal stimulated cortisol levels after ACTH stimulation at the other. We characterised corresponding CYP17A1 enzymatic activities *in vivo*, utilising urinary steroid profiling by gas chromatography-mass spectrometry (GC-MS), and *in vitro,* employing a mammalian overexpression system as well as *in silico* modelling.

## Patients and methods

Informed consent and assent, if applicable, to participate in this study were obtained from patients and parents, respectively. Patients 1–5 were recruited in the study from the *UK Gonadal and Adrenal Inherited Disorders Network* (Institute of Child Health/ Great Ormond Street Hospital Research Ethics Committee; REC number 07/Q0508/24). In patients 6–8, generic consent forms were employed that were designed following guidance published by the Committee on Publication Ethics (COPE) on Journal’s Best Practices Ensuring Consent for Medical Case Reports (www.publicationethics.org).

**Case 1:** The baby (46,XX) was born at 38 weeks gestation by emergency caesarean section due to foetal distress; birth weight was 2530 g (−1.8 SDS). Parents were consanguineous. She was well immediately after birth, not requiring neonatal intensive care support and was discharged on day 2 of life. She was fully bottle-fed, drinking 90 mL term formula every 3–4 h. At 5 weeks, she was assessed for prolonged jaundice and poor weight gain (weight 2640 g, −4 SDS). There was no recorded hypoglycaemia. Her total bilirubin was 194 μmol/L (normal <20) with a conjugated fraction of 41 μmol/L. Investigations for prolonged conjugated jaundice, including an ultrasound of her liver, were normal, but random cortisol was 25 nmol/L and did not increase after stimulation with synacthen (Table 1). Plasma ACTH was highly elevated, suggesting primary adrenal insufficiency. 17OHP was undetectable. Serum potassium and sodium levels were within the normal range. Physical examination showed normal female external genitalia. Findings of urinary steroid profiling by GC-MS (described in results below) were consistent with 17α-hydroxylase deficiency, prompting initiation of glucocorticoid replacement. Without any further interventions, the girl developed well and remained on a dose of 10–12 mg /m^2^/day hydrocortisone.

**Table 1:** Summary of genetic, clinical and hormonal findings. Reference ranges (RR) for hormone measurements are shown in brackets.

	Case 1*	Case 2*	Case 3	Case 4	Case 5^§^	Case 6^§^	Case 7	Case 8
Age at presentation	5 weeks	8 weeks	2 y	17 years	birth	15 years	birth	20 years
Age at investigation	5 weeks	8 weeks	15 y	17 years	12 years	15 years	23 years	20 years
Height, cm			168	164	159.6	161.1	188	158
SDS			0.93	0.1	0.76	−0.96	+1.6	−2.7
Weight, kg	2.62	3.16	49.2	57.7	36.1	48.5	N/A	57.85
SDS	−4	−3.6	−0.55	0.1	0.86	−0.75	N/A	1.3
BMI, kg/m^2^			17.4	21.5	14.2	18.7	N/A	23.1
SDS			−1.12	0.4	−2.1	−0.29	N/A	0.81
Blood pressure, mmHg	N/A	N/A	150/90	129/89	110/80	130/69	120/75	123/71
Na, mmol/L	140	134	140	144	140	N/A	140	142
K, mmol/L	4.5	4.7	3.5	4.2	4.0	N/A	4.2	4.6
Renin/PRA			0.37 ng/mL/h	<5.0 ng/L			1.5 ng/mL/h	1.0 nmol/L/h
RR			0.5–2.6	3.6–20.1			0.5–2.6	0.3–2.2
Aldosterone, pmol/L				<30			0.31	204
RR				50–470			0.1–0.8	<630
Cortisol, nmol/L								
At baseline	<25	<25	136 (>150)	<20	105 (>150)	113 (>150)	375 (>150)	467
60’ ACTH_1–24_	33 (>550)		198 (>550)	30 (>500)	151 (>550)	150 (>550)	621 (>550)	512 (>450)
17OHP, nmol/L	<1 (2–10)	1.5 (2–10)	2.85 (<5)	<0.3 (2–9)	17.4 (<5)	2.7 (<5)	22.1 (0.9–6.6)	28.4 (2–9)
ACTH, pmol/L	85 (<10)		110 (2–10)	15.4 (2–10)	7.7 (2–10)	9.5 (2–10)	4.2 (2–10)	10.2 (2–10)
DHEAS, µmol/L			<0.4 (1.7–10.1)	<0.05	0.4 (1–4)	< 0.4 (1.7–10.1)	0.98 (5.2–12.3)	3.3 (5.2–12.3)
Testosterone, nmol/L				<0.1	2.4 (0.4–9.5)		0.96 (11–35)	6.9 (9.2–55.8)
Oestradiol, pmol/L			<70	<100		43	116 (30–180)	91 (95–228)
LH, U/L			18.4 (0.4–5.7)	33.1	24.4 (0.4–5.7)	2.4 (0.4–5.7)	14 (1–9)	20.0 (1.7–8.6)
FSH, U/L			15.3 (2.7–4.4)	105.1	14.2 (2.7–4.4)	4.3 (2.7–4.4)	21 (1–12)	5.4 (1.5–12.4)
Karyotype	46,XX	46,XX	46,XX	46,XX	46,XY	46,XX	46,XY	46,XY
CYP17A1	Tyr60Ilefs*29 hom	Tyr60Ilefs*29 hom	**Gly111Val/**Pro409Leu	Gly436Arg hom	Phe53/54del hom	Phe53/54del hom	Arg347His/ **Ala398Glu**	**lle371Thr**hom
Parental analysis								
P	N/A	N/A	Pro409Leu	N/A	Phe53/54del	Phe53/54del	N/A	N/A
M	Tyr60Ilefs*29	Tyr60Ilefs*29	Gly111Val	N/A	Phe53/54del	Phe53/54del	N/A	N/A

Novel mutations are highlighted in bold.

^*/§^Siblings.

hom, homozygous; M, maternal allele; SDS, standard deviation score; P, paternal allele; PRA, plasma renin activity.

**Case 2:** The younger sister of case 1 (46,XX) presented to the hospital at 8 weeks of age with failure to thrive. She was born small for gestational age with a birth weight of 2.6 kg at the 42nd week of gestation (−2.7 SDS). Pregnancy and birth were unremarkable. Due to the loss of follow-up with her older sister (case 1), peri-natal investigations were not initiated. She had been discharged on day 2 after normal vaginal delivery. Her weight on admission was 3.18 kg (−3.8 SDS). Her clinical examination was unremarkable, with normal female external genitalia. A random cortisol was undetectable, the 17OHP was low, and her electrolytes were within the normal range (Table 1). There was no recorded hypoglycaemia. Her total unconjugated bilirubin was 22 μmol/L (normal <20). Given the family history, she was started on a maintenance dose of hydrocortisone (10 mg/m^2^/day) and, without any further intervention, she started to gain weight on demand bottle feeds and remained well on ongoing hydrocortisone supplementation. A urinary steroid profile by GC-MS obtained after the initiation of hydrocortisone substitution showed a similar pattern to that of her sister, consistent with 17OHD but with normal concentrations of cortisol metabolites, as would be expected while treated.

**Case 3:** The patient (46,XX) was born at term with a weight of 2200 g (−2.7 SDS) as the third child of non-consanguineous parents of Polish origin. Pregnancy and birth were uneventful. At the age of 2 years, she was diagnosed with hypertension and treatment with β-blockers and calcium antagonists was initiated. During childhood, the girl was frequently hospitalised due to electrolyte disturbances. At the age of 15 years, she was admitted to the paediatric endocrine service for further investigation of delayed pubertal development and hypertension. She presented with proportional body growth (height 168 cm (0.93 SDS), weight 49.2 kg (−0.55 SDS)), lack of pubertal development (Tanner stages PH1, B2) and elevated blood pressure (150/90 mmHg, 3.56/2.98 SDS for height), with normal sodium and low-normal potassium (Table 1). Further hormonal investigations revealed low plasma renin activity (PRA, Table 1); ultrasound investigations excluded renal and cardiac causes of hypertension. Baseline cortisol was low and did not increase after ACTH stimulation; 17OHP was normal and ACTH was elevated (Table 1). Androstenedione and oestradiol (E2) were low with elevated gonadotrophins, indicating hypergonadotropic hypogonadism (Table 1). Pelvic ultrasound showed a prepubertal uterus. There were two hypoechogenic cysts (25 and 19 mm in diameter) within an enlarged right ovary (20.7 cm^3^); the left ovary contained no cysts and was of normal volume (5.7 cm^3^).

Treatment with dexamethasone (0.5 mg/day) was initiated, and after 3 days, the blood pressure normalised to 110/70 mmHg, which was confirmed on further random blood pressure measurements. Two months later, she suffered from acute abdominal pain and an ultrasound examination showed enlargement of both ovarian cysts. Additional treatment of oestradiol (1 mg/day) in combination with GnRH super agonist (triptorelin embonate, 3.75 mg/month) was initiated. Five days later, abdominal pain resolved, and after the second dose of Diphereline, both ovarian cysts regressed completely.

**Case 4:** The girl (46,XX) was referred to the adult endocrine service at the age of 17 years with primary amenorrhoea. She had been under regular endocrine surveillance since birth due to congenital hypothyroidism. She had been treated for essential hypertension with a calcium antagonist from the age of 15 years. Her hormonal investigations were suggestive of primary ovarian insufficiency. An initial MRI pelvis suggested absence of cervix and uterus, with a streaky left ovary and no right ovary visualised. Pubertal induction was commenced by the paediatric endocrine services at the age of 16 years. Further hormonal investigations revealed low plasma renin activity with normal electrolytes, low cortisol at baseline and after ACTH stimulation together with low 17OHP (Table 1). She was started on regular hydrocortisone (11.5 mg/m^2^/day). She remained on levothyroxine, spironolactone and oestradiol patches for pubertal induction. She has developed withdrawal bleeds 1.5 years after initiation of pubertal induction. A transabdominal ultrasound scan of her pelvis, about 1 year after oestrogen supplementation, confirmed the presence of a uterus with oestrogenised endometrium and the presence of ovaries.

**Case 5:** The boy (46,XY) is the fourth child of consanguineous parents of Afghan origin, with no available information on pregnancy and birth. He presented at the age of 12 years with learning difficulties, gynecomastia, micropenis (stretched length 2.5 cm; <3rd centile), glandular hypospadias and cryptorchidism and had a history of orchidopexy for a left undescended testis in the age of 5 years. Tanner stages: G1, PH3, testicular volumes were 1 mL on the right and 4 mL on the left. His blood pressure was normal, serum potassium and sodium levels were within a normal range (Table 1). His serum testosterone was within the lower normal age- and sex-specific range while gonadotrophins were increased, in keeping with compensated hypergonadotropic hypogonadism (Table 1). Baseline cortisol was low and did not increase after i.v. synacthen; 17OHP at baseline was elevated (Table 1).

**Case 6:** The older sister (46,XX) of case 5 presented to the endocrine clinic at the age of 15 years because of the family history and delayed pubertal development. Her previous medical history including pregnancy and birth was unremarkable. She had normal breast development (Tanner B4) but there was an absence of menarche and pubic hair growth. Serum cortisol was low at baseline and did not increase sufficiently after i.v. synacthen (Table 1). Serum 17OHP was 2.7 nmol/L at baseline and increased to 3.5 nmol/L after synacthen stimulation. Baseline DHEAS was undetectable. Serum oestradiol was prepubertal with gonadotrophins at baseline within the pubertal reference range (Table 1). Blood pressure was normal at several random measurements and within the age- and height-adjusted 50th–90th centiles in a 24 h blood pressure measurement.

**Case 7:** The patient (46,XY) was born as the only child of non-consanguineous parents of Caucasian origin in Romania. He presented to the joint endocrine and urology services at the age of 23 years with a wish for phalloplasty and to evaluate his fertility. There are no details available regarding pregnancy, birth and medical history. According to the patient, he was born with a bifid scrotum and an ‘absent phallus’. He was raised as a boy and received multiple testosterone injections during childhood and adolescence. He underwent two unspecified genital surgeries, one at 3 months and another at 3 years of age. At the age of 16 years, bilateral gynecomastia was surgically removed. On examination, there was a single perineal opening with no phallus. Both testicles were small; the left testicle was palpable in the inguinal region; the right was found in a bifid scrotum. Blood pressure was normal when measured randomly and over 24 h. Hormonal investigations showed normal cortisol levels at baseline and after synacthen stimulation (Table 1). His 17OHP was elevated (Table 1), a corresponding 17Preg level was normal (7.9 nmol/L; NR <13.3); the ACTH was within the reference range (Table 1). Sodium, potassium, renin and aldosterone were normal (Table 1). DHEAS and testosterone were both low, with elevated baseline gonadotrophins (Table 1).

**Case 8:** The patient (46,XY) presented to the adult endocrine service with right-sided gynecomastia at the age of 20 years. He was born in Pakistan to consanguineous parents (first cousins). He reported having two operations to correct hypospadias, completed at the age of 7 years before the family moved to the UK. He has had bilateral mammoplasty during adolescence, but the gynaecomastia was refractory to surgical treatment on the right due to residual breast tissue. On examination, there was no genital ambiguity and his external genitalia appeared male with testicular volumes of 9 (left) and 13 mL (right). He had appropriate amounts of pubic and axillary hair. His stretched penile length was 9 cm (below 10th centile). His blood pressure was normal with normal electrolytes and plasma renin activity (Table 1). His 17OHP was mildly elevated and an early morning ACTH was at the upper limit of normal; he had adequate cortisol response after synacthen stimulation (Table 1). Serum testosterone level was slightly below the normal adult male range with elevated LH and normal FSH, indicating hypergonadotropic hypogonadism (Table 1). Imaging studies of his adrenals and testicles did not reveal any pathology; the adrenals were of normal size. He was commenced on testosterone undecanoate 1 g every 12–14 weeks, following sperm cryopreservation. Following testosterone replacement, he experienced an increase in muscle bulk, beard growth, penile growth and disappearance of palpable breast tissue.

### Urinary steroid profiling by gas chromatography-mass spectrometry (GC-MS)

Analysis of urinary steroid metabolite excretion was performed as described previously by a quantitative GC-MS selected ion-monitoring (SIM) method (15). In brief, steroids were enzymatically released from conjugation and, after extraction, chemically derivatised before GC-MS SIM analysis. Steroids quantified included corticosterone metabolites (tetrahydrocorticosterone (THB), 5α-tetrahydrocorticosterone (5αTHB) and tetrahydro-11-dehydrocorticosterone (THA)), the 17-hydroxyprogesterone metabolites 17-hydroxy-pregnanolone (17HP) and pregnanetriol (PT), the pregnenolone metabolite pregnenediol (5PD), the 17-pregnenolone metabolite 5-pregnenetriol (5PT), cortisol metabolites (tetrahydrocortisol (THF), 5α-tetrahydrocortisol (5αTHF), tetrahydrocortisone (THE), cortolones and cortols and androgen metabolites (androsterone (An) and etiocholanolone (Et), DHEA (DHEA)). Additional steroids that are important in the newborn period were quantified in Case 1: 16α-hydroxy-dehydroepiandosterone (16α-OH-DHA), 16α-hydroxypregnenolone 5αTHA, 5αTHF and 6α-hydroxy tetrahydro-11-dehydrocorticosterone (6a-OH-THA).

Following the quantification of steroid metabolites, we calculated substrate metabolite to product metabolite ratios to determine the approximate *in vivo* net activities of CYP17A1: corticosterone over cortisol metabolites (17α-hydroxylase; (THA+THB+5αTHB)/(THF+5αTHF+THE)), and 17-hydroxyprogesterone over androgen metabolites (17,20-lyase; (17HP+PT)/(An+Et) and 5PT/DHEA). The diagnostic ratios were compared to data obtained from a healthy reference cohort (males and females, aged 10–30 years, *n*  = 28).

### Sequencing and analysis of sequence variants

We carried out sequencing analysis for cases 1, 2, 3, 4, 5 and 6 as described previously (16). *CYP17A1* gene analysis in genomic DNA from cases 7 and 8 was performed by genetic service laboratories accredited by the National Health Service, England. Sequencing analysis was performed using Lasergene^®^ software (DNASTAR Inc., Madison, USA), and mutation numbering was carried out referring to the CYP17A1 NCBI reference sequences Chr10(GRCh38) (genomic), NM_000201.4 (cDNA) and NP_000093 (protein). Allele frequencies and REVEL (Rare Exome Variant Ensemble Learner) scores, an ensemble method for the prediction of missense variants (17), were obtained with the gnomAD 3.1.1 software.

### *In silico* analysis

The crystal structure of human CYP17A1 (http://www.rcsb.org/pdb, PDB code 3RUK) was used to analyse the impact of non-synonymous sequence variants on the three-dimensional structure of the CYP17A1 enzyme using the Molsoft ICM Browser Pro Software (Molsoft L.L.C, La Jolla, CA, USA).

### *In vitro* functional studies

The mammalian expression vector pcDNA6-V5-6xHis(B) (Invitrogen) was used for *in vitro* functional assays in HEK293 cells. Plasmids containing either the WT human *CYP17A1* or mutant sequences were synthesised by GenScript Biotech (Amsterdam, The Netherlands). The stop codon in the ORF was removed to add a C-terminal V5-tag. All constructs were confirmed by Sanger sequencing.

Transfection was carried out as described previously (6). A ratio of 2 µg of plasmid DNA to 6 µL of FuGENE^®^HD transfection reagent per six-well was employed. Forty-eight hours after transfection, cells were incubated at 37°C with 1 mL full MEM supplemented with 1000 nmol/L progesterone for 17α-hydroxylase assays or 500 nmol/L 17Preg for 17,20-lyase assays. Substrate concentrations were chosen as they are near the Km of the WT enzyme. Optimal incubation times for obtaining linear conversion rates were assessed for WT and mutants in preceding pilot experiments and were 30 min for 17α-hydroxylase and 60 min for 17,20-lyase.

After incubation, 20 ng of each deuterated progesterone, 17OHP, 17Preg, DHEA, and androstenedione (all Cambridge Isotope Laboratories Inc., Andover, MA) was added to the medium as an internal standard to normalise for extraction efficiency. Steroids were extracted from 500 µL of spiked medium with 1.5 mL methyl tert-butyl ether (MTBE, Sigma–Aldrich) as described previously (18). A Waters Xevo mass spectrometer with ACQUITY ultra performance liquid chromatography™ (UPLC) system was used fitted with an HSS T3, 1.8 µM, 1.2 × 50 mm column (Waters Corporation, Milford, MA). Each steroid was quantified by comparison to a calibration series with respect to their internal standard. Quantitation was completed using TargetLynx 4.1 software (MassLynx 4.1, Waters). Conversion rates of CYP17A1 activities were determined as micrograms per milligram of total protein per minute and expressed as a percentage of substrate conversion defining WT activity as 100%. Data analysis was facilitated and graphically illustrated using the GraphPad Prism software version 5.0 (GraphPad Inc. ).

## Results

### Sequencing analysis

The identified *CYP17A1* sequence variants are summarised in Table 1 (protein level) and Supplementary Table 1 (see section on supplementary materials given at the end of this article) (full HGVS nomenclature). Parental DNA for segregational analysis was available for cases 1–3, 5 and 6. Of the eight sequence variants identified, three have not been reported before (p.Gly111Val, p.Ala398G, p.Ile371Thr). Three sequence variants (p.Pro409Leu, Gly436Arg and p.Tyr60Ile*fs*Lys88X) have been described in clinical case reports but without activity assays (19, 20), while reports of two (p.Phe53/54del, p.Arg347His) have included activity assays (10, 14). All sequence variants have low allele frequencies, and pathogenicity prediction of the missense variants revealed REVEL scores of >0.5, suggesting that they are likely pathogenic (see Supplementary Table 1).

### Urinary steroid profiling

**Figure 1:**
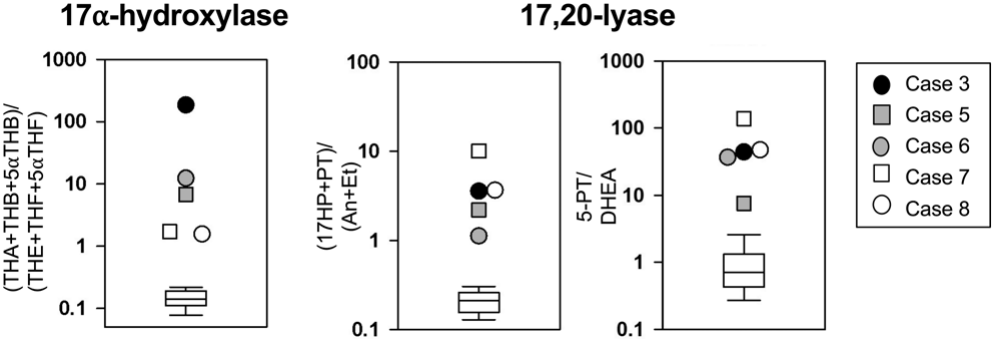
*In vivo* assessment of CYP17A1 17α-hydroxylase and 17,20-lyase activities as indicated by urinary steroid metabolite analysis. Individual diagnostic steroid metabolite ratios in the five 17OHD cases analysed are represented by the symbols as indicated in the legend. Phenotypes with a mild phenotype (cases 7 and 8) are represented as white, intermediate phenotypes (cases 5 and 6) as grey and one case with a severe phenotype (case 3) as black symbols. White box plots represent the interquartile ranges of the reference cohort (healthy males and females, 10–30 years; *n*  = 28), whiskers represent the 5th and 95th percentiles, respectively. For steroid abbreviations please see methods.

All patients had received a diagnosis by urinary steroid profiling in various laboratories. Stored samples were reanalysed for this study, with the exception of neonatal cases 1 and 2 and case 4, who had already been started on hydrocortisone replacement (Fig. 1). All showed a typical pattern, with elevated metabolites of steroids in the mineralocorticoid pathway, elevated progesterone and pregnenolone metabolites (5-PT, PT, and 5-PD), but low androgen metabolites (An and Et) and borderline to very low metabolites of cortisol (THF, 5α-THF, cortolones and cortols). We employed substrate: product ratios reflecting the *in vivo* catalytic activities for CYP17A1 17α-hydroxylase and 17,20-lyase activities. The ratio reflecting 17α-hydroxylase activity ((THA+THB+5aTHB)/(THE+THF+5aTHF)) was most severely impaired in case 3, presenting with a classical phenotype (185.7; NR 0.07–0.18), moderately elevated in cases 5 and 6, presenting with a moderate phenotype (6.8 and 12.3, respectively) and mildly elevated in cases 7 and 8, presenting with a mild phenotype (1.7 and 1.62, respectively) (Fig. 1). We have employed two ratios reflecting CYP17A1 17,20-lyase activities: ((17HP+PT)/(An+Et)) in the Δ4 and 5PT/DHEA in the Δ5 pathway. Both ratios were elevated above the reference range for all cases ((17HP+PT)/(An+Et): 1.15–10.1 (NR 0.09–0.31); 5PT/DHEA: 7.6–138.3 (NR 0.2–3.7); Fig. 1). GC-MS chromatograms for case 1 (Supplementary Fig 1) showed changes consistent with our previous findings for severely affected newborns (21). These comprise increase of THA + 5αTHA/THE + 5αTHE: 37.2 (normal: 0.25) and a very high ratio of 16α-hydroxypregnenolone /16α-OH-DHEA: >200 (normal about unity).

### *In silico* analysis of non-synonymous sequence variants

#### Gly111Val (case 3, classic phenotype)

**Figure 2:**
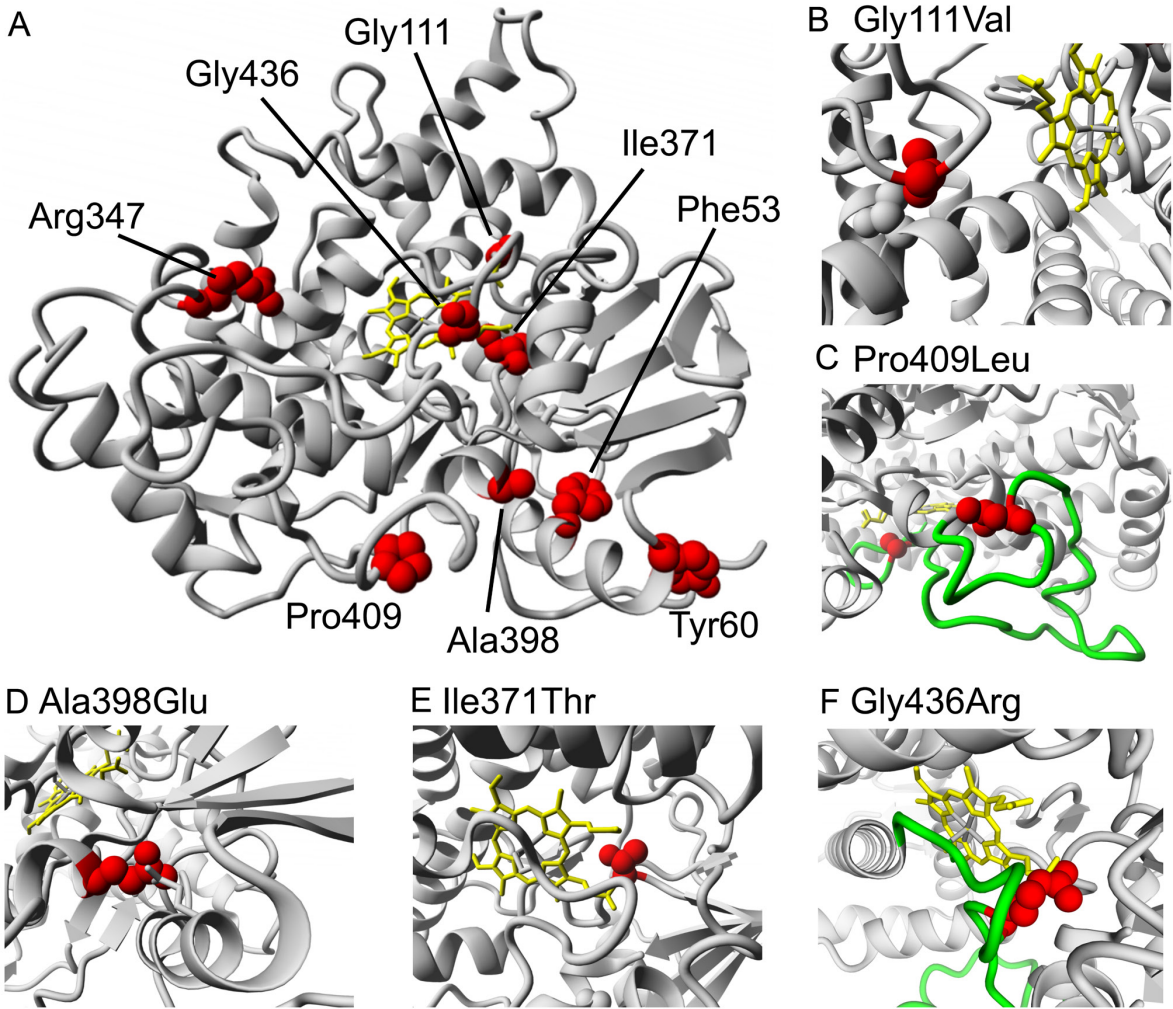
Three-dimensional model of CYP17A1 (RCSB protein databank identifier: 3RUK). Panel A provides an overview of the CYP17A1 model and sequence variants (as WT residues) identified in this case series. Panel B, C, D, E and F magnify the mutated amino acid residues to illustrate the impact of the mutations within their immediate environment.

Glycine 111 is in close proximity to extended helix between threonine 294 and threonine 295, down to a distance of 3.9 Å between Gly111 and Thr294. This Thr294 side chain is shown in ball representation and grey in Fig. 2B. Introducing the bulkier valine instead of Gly111 would result in a steric clash, impairing protein function.

#### Pro409Leu and Gly436Arg (cases 3 and 4, classic phenotypes)

Proline 409 and Glycine 436 are both located in an extended loop structure ranging from asparagine 402 to Glycine 444 (this loop is highlighted in green in Fig. 2C and F). Glycine 436 is a highly conserved residue, embedded in an extended loop structure in close contact with the haeme co-factor. Gly436 is needed for a hairpin loop beginning at Pro434. Exchanging glycine 436 even with the much bigger arginine will certainly disturb folding and interfere with co-factor binding due to steric clashes. The loop region around Pro409 is rather stable according to structural thermal factors. Most likely, it is the unique properties of the amino acid proline that are required to initiate a tight loop including Asp410 and to stiffen the peptide backbone. Even exchanging proline with the similarly sized hydrophobic amino acid leucine will most likely destabilise the protein fold.

#### Ala398Glu (case 7, mild phenotype)

Alanine 398 sits in a relatively polar pocket (Fig. 2D). One of the polar amino acids is glutamine 57, which forms a hydrogen bond with the glycine 77 main chain oxygen. Introducing glutamate instead of alanine 398 may break this hydrogen bond but could offer opportunities for compensating interactions with glutamine 57 or asparagine 395 as the potential binding partners. Hence, this exchange is expected only to mildly influence protein function.

#### Ile371Thr (case 8, mild phenotype)

Isoleucine 371 is in close proximity to arginine 96 (3.4Å) and in spatial proximity to the haem co-factor (Fig. 2E). Threonine is smaller than isoleucine but fits well into the overall shape of isoleucine. An isoleucine to threonine exchange at this buried site is expected not to cause any steric clash and would impair protein function only mildly.

We have used the structural model for a CYP17A1-b5-POR complex (22) to test whether any of the mutations from this study could interfere with redox partner binding. According to this model, none of the mutations described here is likely to interfere with b5 and/or POR binding.

### *In vitro* functional analysis

**Figure 3:**
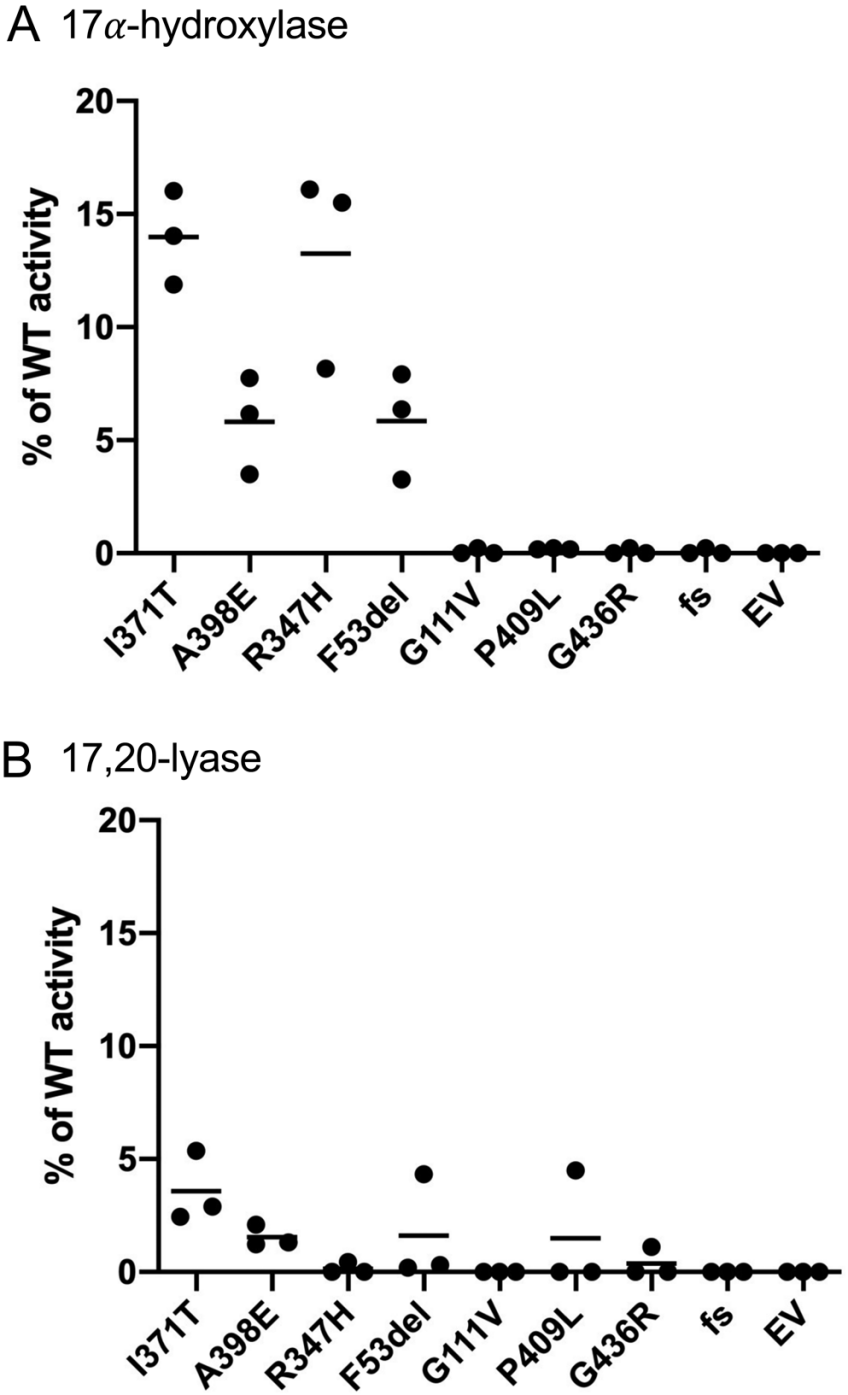
Residual mutant enzyme activity values are shown for the two catalytic activities of CYP17A1. Panel A for the conversion of progesterone (Prog) to 17-hydroxyprogesterone (17OHP), reflecting 17α-hydroxylase activity; panel B reflects the 17,20-lyase activity as assessed by the conversion of 17-hydroxypregnenolone (17Preg) to DHEA. Residual enzyme activity is expressed at the percentage of WT activity, which is defined as 100%. Substrate conversion rate for WT protein was 14.4 ± 0.4 nmol/mg protein/min for Prog and 5.8 ± 1.5 nmol/mg protein/min for 17Preg. All experiments were performed in triplicate in three independent experiments, and each data point represents the median of each triplicate.

All mutants assessed for the residual enzymatic activity of both catalytic reactions of CYP17A1 showed greatly reduced or absent activity in our cell-based *in vitro* system (Fig. 3). Four mutants (p.Ile371Thr, p.Arg347His, p.Ala398Glu and p.Phe53/54del) retain some residual activity on 17α-hydroxylase function with about 10–15% of WT activity (Fig. 3A). Amongst these, the p.Ile371His and the p.Arg347Arg mutations, found in the cases 7 and 8 with a mild phenotype, have the highest residual activity (14.0 ± 1.2% and 13.3% ± 2.6%, respectively) with p.Ala398Glu and p.Phe53/54del retaining 5.8 ± 1.3% and 5.9 ± 1.4%, respectively, of WT enzyme activity. In p.Gly111Val, p.Pro409Leu, p.Gly436Arg and Tyr60Ile*fs*Lys88X, found in cases with severe phenotypes, 17α-hydroxylase activity was abolished in our assay system (Fig. 3A). 17,20-lyase activity was greatly reduced or abolished for all mutant CYP17A1 proteins studied (Fig. 3B). The p.Ile371Thr mutation, found in case 8 with a mild phenotype, retained the highest degree of residual activity (3.6±0.9%). The p.Ala398Glu, p.Phe53/54del and p.Pro409Leu retained residual enzyme activity at 1.5% of wild-type. The residual 17,20-lyase activity is abolished for the p.Arg347His, the p. Gly111Val, the Gly436Arg and the frameshift mutations (Fig. 3B).

## Discussion

Our case series refines the phenotypic spectrum of 17OHD, describing eight patients with inactivating mutations in the *CYP17A1* gene and assessing their functional impact* in vivo* by urinary steroid profiling analysis and *in vitro* by functional studies. This showed that residual CYP17A1 17α-hydroxylase activity is associated with the severity of this condition. Four of our cases present with extreme phenotypes: failure to thrive and conjugated hyperbilirubinemia in our siblings (cases 1 and 2) prompted investigations into adrenal insufficiency caused by a severe homozygous frameshift mutation, and, at the other end of the scale, cases 7 and 8 exhibited a normal cortisol increase in response to ACTH.

The majority of patients with combined 17α-hydroxylase/17,20-lyase deficiency due to severely inactivating *CYP17A1* mutations first present with a lack of pubertal development and low renin hypertension. They may be found to be 46,XX, as in cases 3 and 4, or 46,XY. Patients with milder mutations who are 46,XX may present with less overt pubertal delay and be normotensive, as in case 6, while 46,XY individuals may present at birth with genital ambiguity, although diagnosis may be significantly delayed, as in cases 5, 7 and 8.

Cortisol deficiency in 17OHD is appropriately compensated by activation of the HPA axis to maintain the required level of alternative glucocorticoid, primarily corticosterone, an intermediate in the mineralocorticoid pathway (1, 23, 24). Cases 1 and 2 had increased corticosterone metabolite excretion in the neonatal urinary steroid profile, but failure to thrive, and also conjugated hyperbilirubinaemia in case 1, which may reflect clinical signs of early onset glucocorticoid deficiency. A recent case report of a 46,XX girl harbouring the same frameshift mutation in homozygosity as cases 1 and 2 presented in adolescence with primary amenorrhoea, but details of her neonatal course were not provided (20). Similarly, three Turkish siblings with large-range deletions in *CYP17A1* abolishing enzymatic activity presented with severe hypertension and primary amenorrhoea, but early onset adrenal insufficiency has not been reported by the authors (25). While contributing factors such as stress, infections or sepsis are reported to trigger adrenal crisis in 17OHD, as exemplified in the very first reported case by Biglieri (26), these had been excluded at the time of presentation in our siblings. Both siblings recovered after initiation of physiological doses of hydrocortisone alone, and other causes of failure to thrive and conjugated jaundice were excluded. Importantly, the findings from the urinary steroid profiling analysis were instrumental in excluding other causes of primary adrenal insufficiency (i.e. mutations in *NR5A1*, *StAR*, *CYP11A1* or* HSD3B2*). Multi-steroid profiling by LC-MS/MS has also been shown to be highly effective in detecting CYP17A1 deficiency early (27) but was not available. Given the consanguineous background of our siblings, concomitant mutations in other genes causing adrenal insufficiency might have contributed to the phenotype, a rare possibility that we were not able to address. However, it does appear that phenotypic severity varies even in severely predicted *CYP17A1* genotypes, as exemplified by a report of an adult prepubertal woman with an early truncation of the CYP17A1 protein (homozygous p.Y27X) who did not even develop hypertension (28). Different phenotypes with the same genotype or even family background are recognised in CAH, and genetic variation in other genes or co-factors of steroidogenesis may contribute to this phenotypic variability (29). 'However, on balance, we are not able to conclusively prove that glucocorticoid deficiency due to 17OHD has caused the symptoms observed in these siblings since electrolyte abnormalities and significant hypotension/shock were absent. The clinical signs were subtle and relatively non-specific, nevertheless prompted the clinician to investigate for adrenal pathology'.

Activation of the HPA axis also drives increased production of the corticosterone precursor, 11-deoxycorticosterone (DOC), a weak mineralocorticoid; 18-hydroxylated products of both steroids also have mineralocorticoid activity. This tends to suppress the renin-angiotensin-aldosterone system via blood pressure increase and hypokalaemia. In severe 17OHD, this is insufficient to compensate for the mineralocorticoid excess, resulting in hypertension and hypokalaemia, as in the classic presentations of patients 3 and 4. Milder deficiencies can be compensated, as seen in patients 5–8. Notwithstanding the above, about 10% of patients with severe 17OHD are normokalaemic and normotensive (30). This also suggests that there is considerable phenotypic variability, with perhaps both additional compensatory mechanisms and forms of compensatory failure.

Cases 7 and 8 presented with the clinical and biochemical features of so-called ‘isolated 17,20-lyase deficiency’. This is a rare condition and only around 30 cases have been described in the literature (6, 12, 13, 14, 31, 32). Initial reports provided only clinical and hormonal characterisation without identifying a distinct genetic abnormality in six families with 46,XY DSD and two families with 46,XX patients that presented with lack of pubertal development (reviewed and discussed in (1, 6)). So far, 17 cases of ‘isolated’ 17,20-lyase deficiency with a complete clinical, hormonal, genetic and functional work-up have been reported. Underlying causes were four distinct missense mutations in the *CYP17A1* gene (p.Arg347His; p.Arg347Cys p.Arg358Gln; p.Glu305Gly) (12, 13, 14) and one *POR* missense mutation (p.Gly539Arg) (31). A total of six patients with *CYB5A* mutations have been reported so far (32, 33) and these exhibit the purest form of isolated 17,20-lyase deficiency. CYB5A exclusively supports CYP17A1 17,20-lyase activity, as shown by comprehensive clinical and biochemical phenotyping as well as *in vitro* functional analysis in an affected kindred with the p.His44Lys mutation (6).

Case 7 carried the known missense mutation p.Arg347His on one allele (14) and a novel missense mutation p.Ala398Glu on the other allele. According to our *in vitro* functional analysis, the p.Ala398Glu has only 5.8% residual activity compared to p.Arg347His, which retains 13.3% of WT activity. The finding that our patient has normal stimulated cortisol levels is due to the effect of the milder p.Arg347His mutation. Case 8 carries the novel p.Ile371Thr mutation in homozygosity and also presented with some degree of sex steroid deficiency/primary hypogonadism and normal cortisol levels after ACTH stimulation. It is remarkable that his serum testosterone concentration was within the lower male reference range despite a severe degree of genital ambiguity at birth. This may indicate that testicular testosterone production during embryonic development is more severely affected compared to puberty, which is surprising since accumulating 17OHP due to deficient 17,20-lyase in the foetal testis should feed into the alternative pathway towards dihydrotestosterone generation (4, 34). Further studies on p.Ile371Thr could explore the possibility that this CYP17A1 variant has reduced affinity for 5-pdiol, the substrate of 17,20-lyase in the alternative pathway. Genital ambiguity in 46,XY has a relatively high population prevalence and a molecular basis is not established in most cases (35), so a multi-factorial cause in our patient is possible. The data from our *in vitro* functional analysis concur with the clinical findings: the 17α-hydroxylase activity was 14%, a similar level to the p.Arg347His mutation, but it also retained some residual 17,20-lyase activity.

At the time of assessment, 46,XY patients 7 and 8 had high serum levels of 17OHP, as did case 5, who had a much higher level than his 46,XX sibling, case 6. This likely reflects predominantly enhanced testicular 17OHP production under LH hyperstimulation. This is supported by the urine steroid data, which showed increases above normal of the 17OHP metabolites 17HP and PT but not of 11-oxopregnanetriol, the major metabolite of 21-deoxycortisol and a marker of adrenal 17OHP excess. This indicates a predominantly testicular origin for the 17OHP. Prepubertal XY patients with 17OHD have been reported to show increased 17OHP after hCG stimulation but not after synacthen.

To our knowledge, cases 7 and 8 have the mildest ever reported phenotype for disease-causing mutations in *CYP17A1* and are probably closest to what was previously described as ‘isolated’ 17,20-lyase deficiency in the context of 17OHD. A unifying characteristic of all individuals with ‘isolated’ 17,20-lyase deficiency is sex steroid deficiency, with hormonal measurements confirming diminished adrenal and gonadal androgen synthesis. All previously reported patients with an apparent isolated 17,20-lyase deficiency with underlying *CYP17A1* or *POR* mutations have shown impairment of cortisol production, with insufficient responses to ACTH stimulation (6). In contrast, cases 7 and 8 are the first reported 17OHD patients who have normal ACTH-stimulated cortisol levels. Nevertheless, urinary steroid analysis demonstrated impairment of 17α-hydroxylase activity. Compared to the ratios we have obtained from patients with classical 17OHD or partial combined 17α-hydroxylase/17,20-lyase deficiency (cases 5 and 6), their corticosterone/cortisol metabolite ratios are lower, suggesting higher residual *in vivo* CYP17A1 17α-hydroxylase activity in milder cases. Thus, a precursor/product ratio of corticosterone over cortisol metabolites reflecting *in vivo* 17α-hydroxylase activity correlates well with the severity of the disease and may help to predict the phenotype (Fig. 1). This finding is in accord with two previous studies employing GC-MS steroid metabolome analysis in 17OHD subjects with severe (36) and milder (37) disease manifestation. Interestingly, the diagnostic ratios reflecting CYP17A1 17,20-lyase activity in our patients are higher in milder cases (cases 7 and 8) suggesting severe impairment (Fig. 1). Severe 17α-hydroxylase attenuation in more severe disease results in the reduction of 17-hydroxylated metabolites that build the numerator for both ratios. Hence, neither 17,20-lyase ratios reflect the *in vivo* residual activity in CYP17A1 due to concomitant 17α-hydroxylase inhibition.

In our functional analysis, we have not performed enzyme kinetic studies in WT and mutant protein to determine Vmax and Km, thus the reported residual activity at a fixed concentration, around the Km of the substrates of the WT protein, is a combination of both effects. This is a limitation of our work, however, enables the determination of the disease-causing effects of the variants and characterises their differential impact on 17α-hydroxylase and 17,20-lyase activities.

In summary, our case series illustrates the broad phenotypic variability of CYP17A1 deficiency and, importantly, expands both ends of this spectrum. These range from early presentation to isolated sex steroid deficiency with normal stimulated cortisol production, respectively. Urinary steroid profiling by mass spectrometry represents a powerful diagnostic tool not only in establishing the diagnosis but also in predicting the severity of the phenotype (38).

## Supplementary Material

Supplementary Figure 1

Supplemental Table 1: Human Genome Variation Society (HGVS) nomenclature, allele frequencies (according to GnomAD database: www.gnomad.broadinstitute.org), REVEL (Rare Exome Variant Ensemble Learner) score for the prediction of pathogenicity of missense variants (17), and accession numbers of the Human Genome Mutation Database (HMGD; www.hgmd.cf.ac.uk), if available, for all identified sequence variants in this case series. A higher REVEL score (range 0-1) suggests a higher likelihood of pathogenicity.

## Declaration of interest

The authors declare that there is no conflict of interest that could be perceived as prejudicing the impartiality of this study.

## Funding

This work was supported by the Medical Research Council UK (Research Fellowship G1001964, to J I, and Program Grant 0900567, to W A), the European Society for Paediatric Endocrinology (Research Fellowship, to J I), the Academy of Medical Sciences (Starter Grant for Clinical Lecturers SGL020/1013, to J I), and the National Institute of Health Research (Academic Clinical Lectureship to J I). W A receives support from the National Institute for Health Research (NIHR) Birmingham Biomedical Research Centre at the University Hospitals Birmingham NHS Foundation Trust and the University of Birmingham (Grant BRC-1215-20009). The views expressed are those of the authors and not necessarily those of the NIHR or the Department of Health and Social Care, UK. The funders of the study had no role in the: design and conduct of the study; collection, management, analysis and interpretation of the data; preparation, review or approval of the manuscript; decision to submit the manuscript for publication.
