# Supplementary figures and images for: The broad phenotypic spectrum of 17α-hydroxylase/17,20-lyase (CYP17A1) deficiency: a case series
Source: Eur J Endocrinol. 2021 Sep 15;185(5):729–41. doi: 10.1530/EJE-21-0152 (PMC8558848; doi:10.1530/EJE-21-0152)

Suppl. Fig. 1

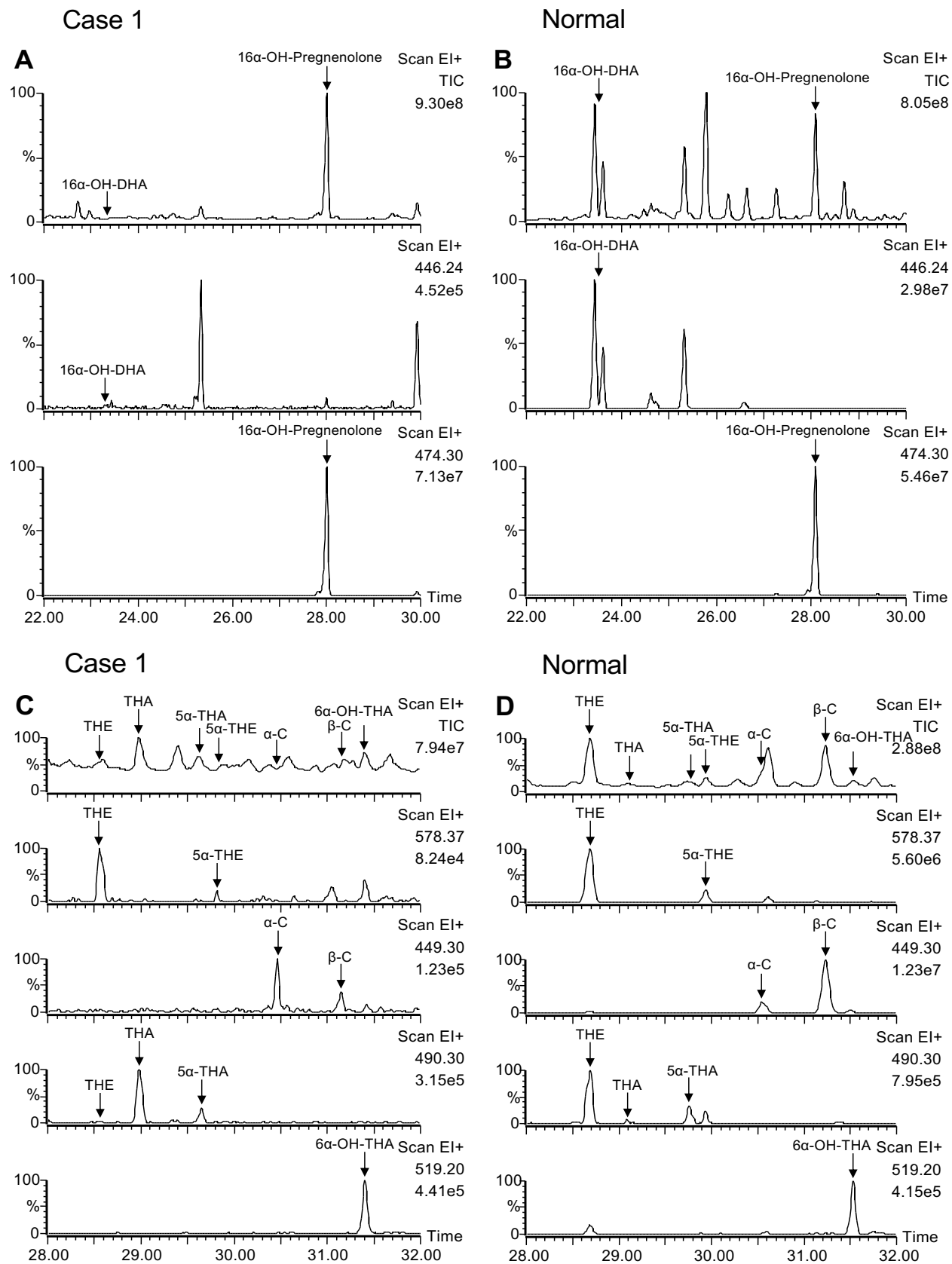

Supplement: Supplementary Figure 1 [file supplementary_figure_1.pdf]
