# Supplementary material for: The broad phenotypic spectrum of 17α-hydroxylase/17,20-lyase (CYP17A1) deficiency: a case series
Source: Eur J Endocrinol. 2021 Sep 15;185(5):729–41. doi: 10.1530/EJE-21-0152 (PMC8558848; doi:10.1530/EJE-21-0152)
Supplement: Supplemental Table 1: Human Genome Variation Society (HGVS) nomenclature, allele frequencies (according to GnomAD database: www.gnomad.broadinstitute.org), REVEL (Rare Exome Variant Ensemble Learner) score for the prediction of pathogenicity of missense variants (17), and accession numbers of the Hu [file supplementary_table_1.pdf]

Supplemental Table 1: Human Genome Variation Society (HGVS) nomenclature, allele frequencies (according to GnomAD database: www.gnomad.broadinstitute.org), REVEL (Rare Exome Variant Ensemble Learner) score for the prediction of pathogenicity of missense variants (17), and accession numbers of the Human Genome Mutation Database (HMGD; www.hgmd.cf.ac.uk), if available, for all identified sequence variants in this case series. A higher REVEL score (range 0-1) suggests a higher likelihood of pathogenicity.

**Protein level (NP_000093)**	p.(Tyr60Ilefs*29)	p.(Pro409Leu)	p.(Gly111Val)	p.(Gly436Arg)	p.(Phe54del)	p.(Arg347His)	p.(Ala398Glu)	p.(lle371Thr)
**cDNA level (NM_000201.4)**	c.177dupA	c.1226C>T	c.332G>T	c.1306G>A	c.160_162del	c.1040G>A	c.1193C>A	c.1112T>C
**gDNA level [Chr10(GRCh38)]**	g.102837185dup	g.102831525G>A	g.102835358C>A	g.102830923C>T	g.102837200_102837202del	g.102832610C>T	g.102831558G>T	g.102832538A>G

**Allele Frequency (GnomAD)**	0.0004%	0.0004%	Not found	0.0018%	0.0024%	0.0016%	Not found	0.0025%
**REVEL Score**	NA	0.816	0.578	0.931	NA	0.704	0.617	0.585
**Listed in HGMD database (accession number)**	Yes (CD185228)	Yes (CM185227)	No	Yes (BM1586049)	Yes (CD890143)	Yes (CM960476)	No	No
